# Time elapsed after contrast injection is crucial to determine infarct transmurality and myocardial functional recovery after an acute myocardial infarction

**DOI:** 10.1186/s12968-015-0139-8

**Published:** 2015-05-30

**Authors:** José F. Rodríguez-Palomares, José T. Ortiz-Pérez, Daniel C. Lee, Chiara Bucciarelli-Ducci, Paula Tejedor, Robert O Bonow, Edwin Wu

**Affiliations:** Department of Cardiology, Institut de Recerca (VHIR), Hospital Universitari Vall d´Hebron, Universitat Autònoma de Barcelona, Barcelona, Spain; Department of Medicine, Division of Cardiology, Bluhm Cardiovascular Institute, Northwestern Memorial Hospital, Northwestern University Feinberg School of Medicine, Chicago, IL USA; Departments of Medicine and Radiology, Northwestern University Feinberg School of Medicine, Chicago, IL USA; Department of Cardiology, Hospital Universitari Vall d’Hebron, Paseo Vall d’Hebron 119-129, Barcelona, 08035 Spain; Bristol Heart Institute, NIHR Bristol Cardiovascular Biomedical Research Unit, University of Bristol, Bristol, UK

**Keywords:** Cardiovascular magnetic resonance, Infarct size, Microvascular obstruction, Gadolinium, Dynamic change

## Abstract

**Background:**

In acute myocardial infarction (MI), late Gadolinium enhancement (LGE) has been proposed to include the infarcted myocardium and area at risk. However, little information is available on the optimal timing after contrast injection to differentiate these 2 areas. Our aim was to determine in acute and chronic MI whether imaging time after contrast injection influences the LGE size that better predicts infarct size and functional recovery.

**Methods:**

Subjects were evaluated by cardiovascular magnetic resonance (CMR) the first week (n = 60) and 3 months (n = 47) after a percutaneously revascularized STEMI. Inversion-recovery single-shot (ss-IR) imaging was acquired at multiple time points following contrast administration and compared to segmented inversion-recovery (seg-IR) sequences. Inversion time was properly adjusted and images were blinded, randomized and measured for LGE volumes.

**Results:**

In acute MI, LGE volume decreased over several minutes (p = 0.005) with the greatest volume occurring at 3 minutes and the smallest at 25 minutes post-contrast injection; however, LGE volume remained constant over time in chronic MI (p = 0.886). Depending on the imaging time, in acute phase, a change in the transmurality index was also observed. A transmural infarction (>75%) at 25 minutes better predicted the absence of improvement in the wall motion score index (WMSI), a higher increase in left ventricular volumes and a lower ejection fraction compared to 10 minutes.

**Conclusions:**

A change was observed in LGE volume in the minutes following contrast administration in acute but not in chronic MI. Infarct transmurality 25 minutes post-contrast injection better predicted infarct size and functional recovery at follow-up.

## Background

Cardiovascular magnetic resonance (CMR) utilizing the late Gadolinium enhancement (LGE) technique is a highly accurate method for characterizing reversible and irreversible myocardial injury to obtain information on myocardial viability in both acute and chronic myocardial infarction [1,2]. Studies in a canine model with 2-day-old reperfused infarcts showed that regions of LGE directly corresponded to regions of infarcted tissue assessed by triphenyltetrazolium chloride staining (TTC) [3]. Gadolinium-based contrast agents using doses of ≥ 0.2 mmol/kg body weight have subsequently been shown to be effective in the detection and assessment of both acute and chronic myocardial infarction (MI) in large cohorts and multicenter clinical studies [4].

However, early studies in animal models conducted at different minutes after the administration of contrast in reperfused acute MI showed that the area of LGE could potentially overestimate the true extent of infarction [5–7]. Moreover, studies in humans performed after an acute MI showed that some transmural regions of LGE recover function over time [8,9]. These findings suggested that Gadolinium enhancement can potentially occur in both reversibly- and irreversibly-injured myocardium. A recent study has confirmed that early enhancement 2 minutes after contrast injection depicted both ischemically-injured but salvaged myocardium and infarcted myocardium, whereas LGE 10–15 minutes after contrast injection revealed only the infarcted myocardium [10].

Rather than conclude that Gadolinium overestimates acute infarct size, it is more challenging to understand the kinetics of Gadolinium contrast enhancement and its wash-out to determine why Gadolinium can show both the penumbra of the area at risk and the core of the LGE that represents the infarct [11].

The aims of this study were to serially investigate differences in LGE and MVO sizes over time after contrast injection in acute and chronic infarcts and also to determine the timing post-contrast that better predicts myocardial functional recovery, left ventricular volumes and ejection fraction.

## Methods

### Patients

Between January 2006 and July 2008, 72 patients admitted to the coronary care unit following an ST-segment elevation acute myocardial infarction (STEMI) successfully reperfused through percutaneous coronary intervention (PCI) were prospectively enrolled. Patients were included if they fulfilled the criteria of documented acute STEMI with [1] chest pain for more than 30 minutes, [2] at least 1.0 mm ST-segment elevation in two contiguous leads, and [3] elevated creatinine phosphokinase MB (CPKMB) isoenzyme or troponin I levels more than two times higher than normal. Patients with a known history of previous acute MI, PCI or coronary artery bypass surgery, frequent and recurrent ventricular arrhythmias, unstable hemodynamics (NYHA class IV), or contraindications to CMR (such as pacemakers, defibrillator) were excluded. A total of 60 patients were finally included in the study. The protocol was approved by the Ethics Committee of our institution, and all patients signed their informed consent.

### Cardiovascular Magnetic Resonance

All CMR studies were performed with a 1.5 T clinical scanner (Sonata or Avanto scanner Siemens, Erlangen, Germany) using a phased-array cardiac receiver coil. Retrospectively electrocardiogram-gated breath-hold short-axis cine views were performed to quantify volumes and ejection fraction (SSFP sequences; slice thickness: 6 mm; space between slices 67%; matrix: 256x256: field of view: 300-370 mm; temporal resolution <50 ms). Additional 2-chamber, 3-chamber and 4-chamber views were also obtained. LGE images were acquired at identical slice positions to the cine images after the administration of 0.2 mmol/kg of body weight Gadolinium-DTPA (Gd-DTPA) (Berlex, Montville, NJ, USA). A single-shot inversion recovery (ss-IR) steady-state free precession sequence [12] was acquired at repeated time points, 1, 3, 5, 7, 10, 15, 20 and 25 minutes following contrast administration (Matrix 192 × 144, voxel size 2.4 × 1.8 × 6 mm, TE 1.08 ms, TR 700 ms, flip angle 50°; and the bandwidth 1180 Hz/pixel). The cutoff value of 25 minutes was established based on previous experimental studies by Oshinski et al. [5]. Inversion time (IT) was appropriately set at any time point to null normal myocardium[13]. In addition, a segmented inversion-recovery (seg-IR) gradient-echo sequence was acquired starting at 10 min after contrast administration (Matrix 256 × 197, voxel size 2.0 × 1.6 × 6 mm, TE 4.91 ms, TR 700 ms, flip angle 30°; and the bandwidth 140 Hz/pixel) [2,13,14].

#### Image analysis

Quantitative analyses of left ventricular (LV) mass, end-diastolic volume (EDV), end-systolic volume (ESV) and ejection fraction (EF) were performed by manually tracing the epicardial and endocardial borders as previously described [15]. Volume indices were calculated by dividing the EDV or ESV by body surface area. Contractility was analyzed and the wall motion score index estimated (WMSI) [2].

For objective quantification of LGE and MVO, a reference region of interest (ROI) was placed in remote myocardium. The signal intensity threshold indicating LGE was imposed 2 standard deviations (SD) above the mean intensity of the reference ROI on each of the contrast-enhanced CMR tomograms and summed, as previously described [2,13,14]. Microvascular obstruction (MVO) was defined as the low intensity core within an area of LGE. *Early MVO* were considered if they were present 1 minute after contrast administration [16] and *late MVO* when seen 10 min later [17], on single-shot images. Areas of MVO were included in the total LGE area [17,18]. The summed area was multiplied by the specific gravity of myocardium to obtain the LGE volume. Relative LGE volume was the percentage of total LV mass (% of LV mass) that presented LGE. To assess the kinetics of Gd-DTPA, all images obtained by the ss-IR (*acute and chronic CMRs*) were blinded, randomized and measured for LGE volumes; 990 stacks of images were analyzed. Dynamic changes in LGE were evaluated with the use of different parameters: LGE volume, relative LGE volume, LGE transmurality and LGE lateral extension.

LGE transmurality was expressed as the mean percentage of transmurality of the LGE in all affected slices at each time point. Transmurality in each slice was calculated by dividing the LGE mass by the total mass of the affected myocardium in each segment [19,20].

LGE lateral extension was determined by calculation of the infarct endocardial surface area (Infarct-ESA), as previously described [21].

Further, single-shot stacks of images in the *acute and chronic* CMRs were analyzed using different standard deviations (2, 3, 4 and 5) over remote signal intensity to establish the effect of different thresholds on LGE volume.

Signal intensity was obtained in the hyperenhanced area, the remote myocardium and background noise for calculation of the signal-to-noise and contrast-to-noise ratios [12].

In a subset of 56 stacks of single-shot images including a mix of acute and chronic CMRs, interobserver and intraobserver reproducibility was performed.

Finally, the LGE volume obtained with seg-IR sequences was measured and compared with the volume obtained by the ss-IR at the same slice position and same time point in order to compare both sequences.

### Statistical analysis

Continuous demographic variables were expressed as mean ± standard deviation. The Kolmogorov-Smirnov test was used to evaluate the normality distribution of variables. Inter-group differences for continuous parameters were assessed by Student's *t*-test if they presented a normal distribution or ANOVA with Bonferroni correction for multiple comparisons, and Mann–Whitney *U* test if they did not present a normal distribution. For categorical variables, general characteristics of the sample were assessed by percentages (chi-square test). Interobserver and intraobserver variabilities were calculated using the intraclass correlation coefficient. Agreement between ss-IR and seg-IR sequences was examined using the intraclass correlation coefficient and Bland–Altman analysis.

A two-tailed P value < 0.05 was considered statistically significant. SPSS 19.0 software version (IBM SPSS Statistics, Chicago, Illinois, USA) was used for the analysis.

## Results

### Baseline characteristics

The clinical and angiographic characteristics of patients are shown in Table 1. All subjects had successful reperfusion with PCI within 24 hours of admission. CMR studies were performed within 1 week (*Acute CMR*, mean: 2.6 ± 1.5 days; range: 1–6) and approximately 4 months (*Chronic CMR*, mean: 122.1 ± 29.7 days; range: 78–195 days) after admission.

**Table 1:** Clinical characteristics for all subjects (n = 60)

**Risk factors**	
Age (years)^1^	57 ± 10
Male	49 (81.67%)
Diabetes mellitus	3 (5%)
Hypertension	25 (41.67%)
Hypercholesterolemia	30 (50%)
Tobacco use	30 (50%)
**Clinical Data**	
Symptom-to-balloon (*median*) (min)	197
Door-to-balloon (*median*) (min)	85.5
Maximum total CK MB (IU/L)^1^	243.65 ± 239.64
Maximum total Troponine T (ng/L)^1^	53.43 ± 80.65
**Angiographic findings**	
Left anterior descending IRA	33 (55%)
Left circumflex IRA	6 (10%)
Right coronary IRA	21 (35%)
*TIMI pre-procedure*	
TIMI 0	43 (71.67%)
TIMI 1	10 (16.67%)
TIMI 2	5 (8.33%)
TIMI 3	2 (3.33%)
*TIMI post-procedure*	
TIMI 2	7 (11.67%)
TIMI 3	53 (88.33%)
*Rentrop Scale*	
0	22 (36.67%)
1	16 (26.67%)
2	15 (25%)
3	7 (11.67%)
**MRI Data**	
*Acute MRI*	
End-diastolic volume (mL)^1^	154.35 ± 30.72
End-systolic volume (mL)^1^	85.14 ± 29.55
Ejection Fraction (%)^1^	45.83 ± 11.47
Infarct mass (gr)^1^	24.12 ± 16.15
*Chronic MRI*	
End-diastolic volume (mL)^1^	161.65 ± 30.73
End-systolic volume (mL)^1^	79.12 ± 28.13
Ejection Fraction (%)^1^	52.16 ± 10.82
Infarct mass (gr)^1^	13.74 ± 10.46
**Medication use**	
IIB-IIIA inhibitor	58 (96.67%)
Antiplatelet (aspirine and/or clopidogrel)	58 (96.67%)
β-Blocker	58 (96.67%)
ACE inhibitor	51 (85%)
Statin	56 (94%)

Data expressed as mean (percentages). ^1^Data expressed as mean and standard deviation.

### Kinetics of Gd-DTPA by ss-IR sequences: *acute versus chronic CMR*

#### Inversion time, signal-to-noise and contrast-to-noise ratios

**Fig. 1:**
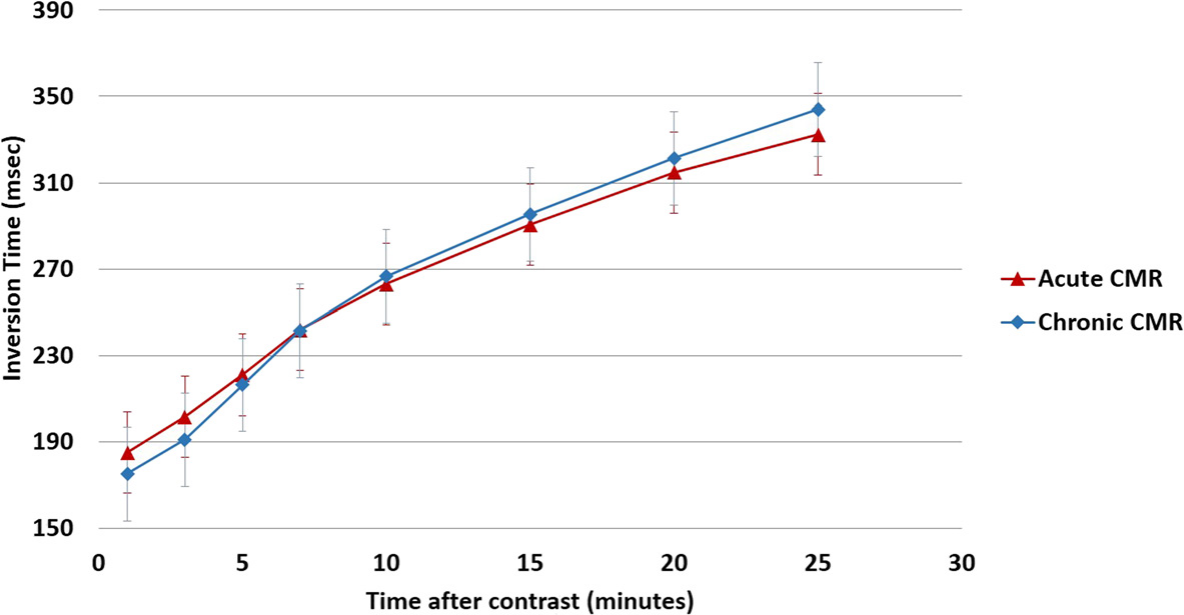
Relationship between the inversion time in the acute and chronic CMR over time after contrast administration

A significant increase in IT was observed after the bolus contrast injection; however, no differences were observed in IT at each time point in the *acute* compared to the *chronic* CMRs (p > 0.1 for all comparisons) (Fig. 1).

**Fig. 2:**
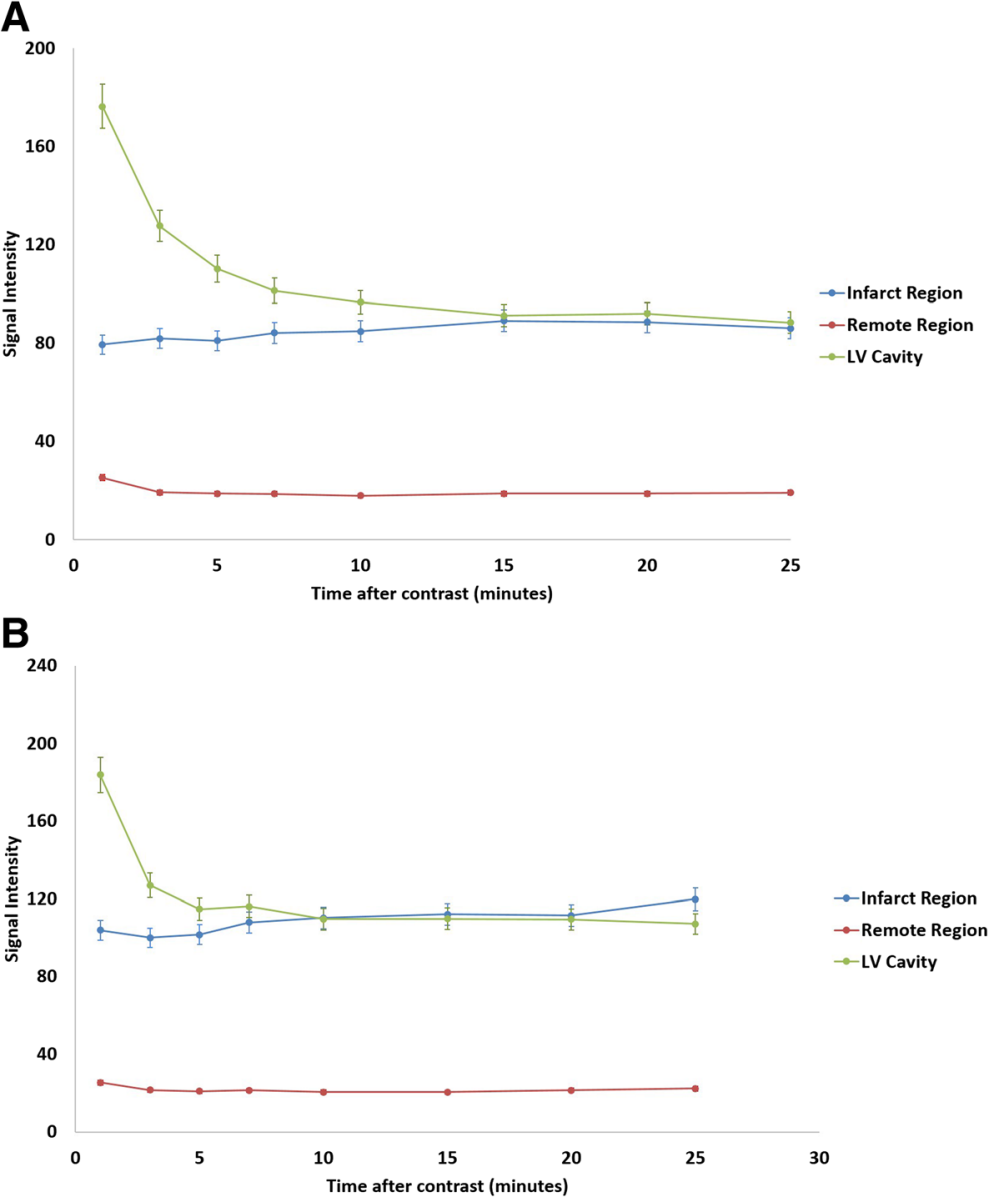
Relationship between the signal intensity in the left ventricular (LV) cavity, the infarcted and remote regions and the time after bolus injection in the acute **(2A)** and chronic CMR **(2B)**

In *acute* CMR, after the injection of Gd-DTPA, the mean signal intensity in the infarct region was 495.45 ± 8.35% higher than the remote region. Signal intensity increased slightly over several minutes, but was not statistically significant (p = 0.727) (Fig. 2A). Thus, no significant change occurred in the signal-to-noise (p = 0.137) and contrast-to-noise ratios (p = 0.140). Similar findings were observed in the signal-to-noise and contrast-to-noise ratios in *chronic* CMR, with no differences in their values, p = 0.873 and p = 0.706, respectively (Fig. 2B).

#### LGE volume and relative LGE volume

In the analysis of infarct size, an intraclass correlation coefficient of 0.94 was found (95% confidence interval (CI): 0.88-0.96; p < 0.0001) for interobserver and 0.98 (95% CI: 0.96-0.99; p < 0.0001) for intraobserver variabilities.

**Fig. 3:**
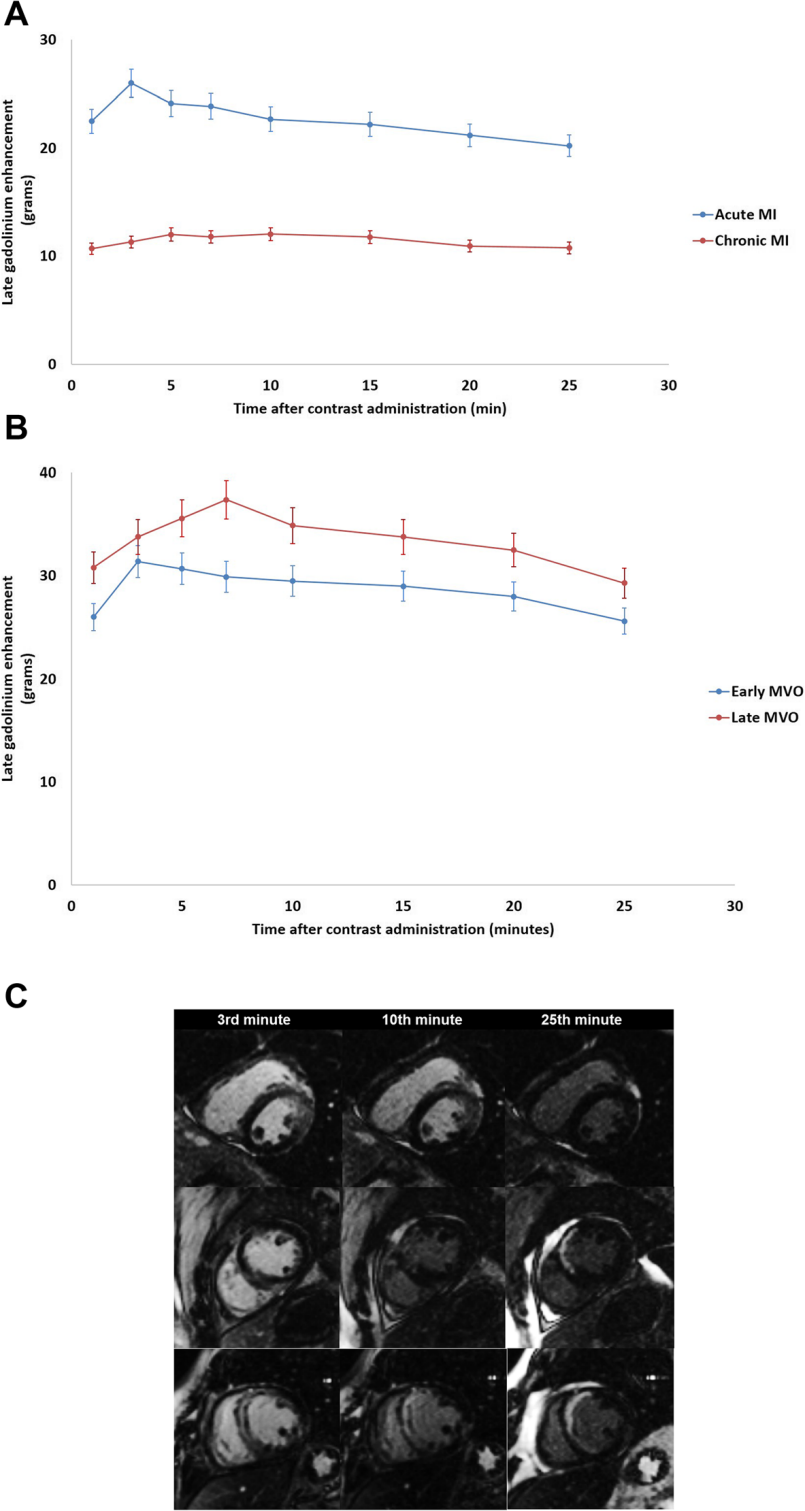
Dynamic changes of late Gadolinium enhancement volume over time after contrast injection in acute and chronic CMR **(3A)** and also in the presence of early and late microvascular obstruction **(3B)**. Representative images of the changes in late Gadolinium and microvascular obstruction volumes in 3 patients (each patient is represented in a row) at different time points (3, 10 and 25 minutes after the administration of the contrast) **(3C)**

Using ss-IR sequences, a significant dynamic change in LGE volume (p = 0.005) and relative LGE volume (p = 0.005) over several minutes after contrast administration was observed. The largest increase in LGE volume was between the 1st and 3rd minutes, and LGE volume subsequently decreased in *acute CMR*. A decrease of 4.91 ± 9.08 grams, 22.20% relative reduction between the 3rd and 25th minutes, p = 0.001 was observed (Table 2, Fig. 3A). Representative images are shown in Fig. 3C. Nevertheless, LGE volume remained constant over time in *chronic CMR* with no significant differences after contrast administration (p = 0.886) (Table 2, Fig. 3A). There were no differences in the mean infarct mass regarding the day at which the CMR was performed (p = 0.066). However, this dynamic pattern was observed regardless of the day at which the CMR was performed.

**Table 2:** LGE and MVO volumes at different time points after the administration of the contrast in acute and chronic CMR

Minutes	Acute LGE volume (grams)	Acute MVO volume (grams)	Chronic LGE volume (grams)
1st	22.49 ± 15.13	6.31 ± 10.92	10.68 ± 7.48
3rd	25.99 ± 14.64	5.07 ± 10.46 (19.51%)*	11.29 ± 6.86
5th	24.13 ± 14.32	3.98 ± 8.26 (36.93%)*	11.99 ± 7.93
7th	23.86 ± 14.43	3.50 ± 8.20 (44.53%)*	11.77 ± 8.39
10th	22.67 ± 13.77	2.77 ± 7.42 (56.10%)*	12.02 ± 8.17
15th	22.20 ± 13.61	2.05 ± 6.40 (67.52%)*	11.75 ± 7.81
20th	21.19 ± 12.62	1.81 ± 6.66 (71.32%)*	10.92 ± 7.12
25th	20.22 ± 12.41	0.79 ± 2.67 (87.32%)*	10.75 ± 7.21

Data expressed as mean and standard deviation. *Data expressed as percentage of reduction.

*Early MVO* was seen in 35 patients (58%) and *late MVO* in 16 (27%), all of whom also had *early* MVO. MVO was not present in the *chronic* CMR studies. A significant decrease was observed in MVO volume (p = 0.005) with the greatest volume 1 minute after contrast administration and the smallest after 25 minutes (Table 2). Dynamic changes in LGE volume also occurred regardless of the presence of *early or late MVO*. Patients with *late MVO* presented slower wash-in of Gd-DTPA with a maximum LGE volume at 7 minutes post-contrast injection compared with the 3 minutes observed in patients with *early MVO* (*early MVO:* p = 0.022 and *late MVO*: p = 0.046) (Fig. 3B).

#### LGE transmurality and LGE lateral extension

**Fig. 4:**
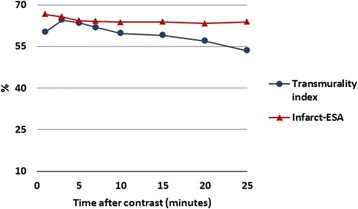
Dynamic changes of the transmurality index and the infarct-ESA over time after contrast administration

In *acute* CMR, a drop in LGE transmurality was observed over time with an increase for the first 3 minutes and with a subsequent decrease (following the same pattern as LGE volume over time) (p = 0.05). However, the infarct-ESA remained stable over time, thereby suggesting that the change in LGE size was due to a decrease in its transmurality rather than in its lateral extension (p = 0.981) (Fig. 4).

### Different sequences to assess contrast enhancement

#### LGE size: ss-IR versus seg-IR sequences

In *acute* CMR studies, each image obtained by the seg-IR sequence was compared with its homologous ss-IR at the same slice position and same time point. Mean LGE size by ss-IR (3.57 ± 1.96 g) did not differ statistically from the seg-IR (3.48 ± 1.91 g), p = 0.72. Intraclass correlation coefficients between ss-IR and seg-IR were 0.92 (95% CI: 0.89-0.94; p < 0.0001). Bland-Altman analysis showed a bias of 0.09 grams of LGE size (95% CI: 0.05, 0.23).

#### LGE volume and different standard deviations over remote signal intensity

**Fig. 5:**
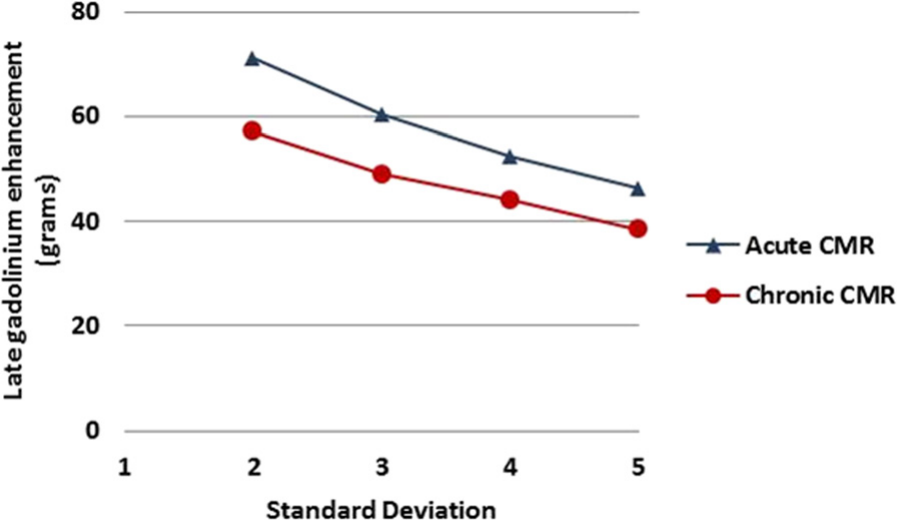
Late Gadolinium enhancement volume obtained by single-shot sequences analyzed using different thresholds (2, 3, 4 and 5 standard deviation over remote signal intensity) in acute and chronic CMR

The LGE size obtained by ss-IR sequences was analyzed using different thresholds (2, 3, 4 and 5 SD over remote signal intensity) and no manual planimetry correction. A reduction in mean LGE size was observed when the SD was increased in both *acute* (p = 0.019) and *chronic* settings (p = 0.039) (Fig. 5). The slopes of both curves were similar, which suggested that increasing the threshold and thus changing the method of quantifying LGE size would result in smaller LGE sizes in the same proportion for acute and chronic settings.

### Effect of timing of LGE measurement to estimate the transmurality index and the recovery of left ventricular function

A dynamic change on the transmural index and the number of patients at a certain degree of transmurality (Table 3) was observed. Thus, 10 min post-contrast, 15 patients (25%) presented a transmural index ≥ 75%, however, only 7 patients (11.67%) at 25 minutes.

**Table 3:** Change of the transmurality index over time (in minutes) after the administration of the contrast in acute CMR

Transmurality index				
**Time**	**<25%**	**25-50%**	**51-75%**	**>75%**
**1 min**	8 (13.33%)	13 (21.67%)	21 (35%)	18 (30%)
**3 min**	2 (3.33%)	10(16.67%)	24 (40%)	24 (40%)
**5 min**	2 (3.33%)	9 (15%)	32 (53.33%)	17 (28.33%)
**7 min**	1 (1.67%)	12 (20%)	30 (50%)	17 (28.33%)
**10 min**	1 (1.67%)	15 (25%)	29 (48.33%)	15 (25%)
**15 min**	1 (1.67%)	14 (23.33%)	32 (53.33%)	13 (21.67%)
**20 min**	1 (1.67%)	19 (31.67%)	30 (50%)	10 (16.67%)
**25 min**	1 (1.67%)	21 (35%)	31 (51.67%)	7 (11.67%)

Data expressed as number of patients with a certain degree of transmurality index (percentages). Min = minutes.

**Fig. 6:**
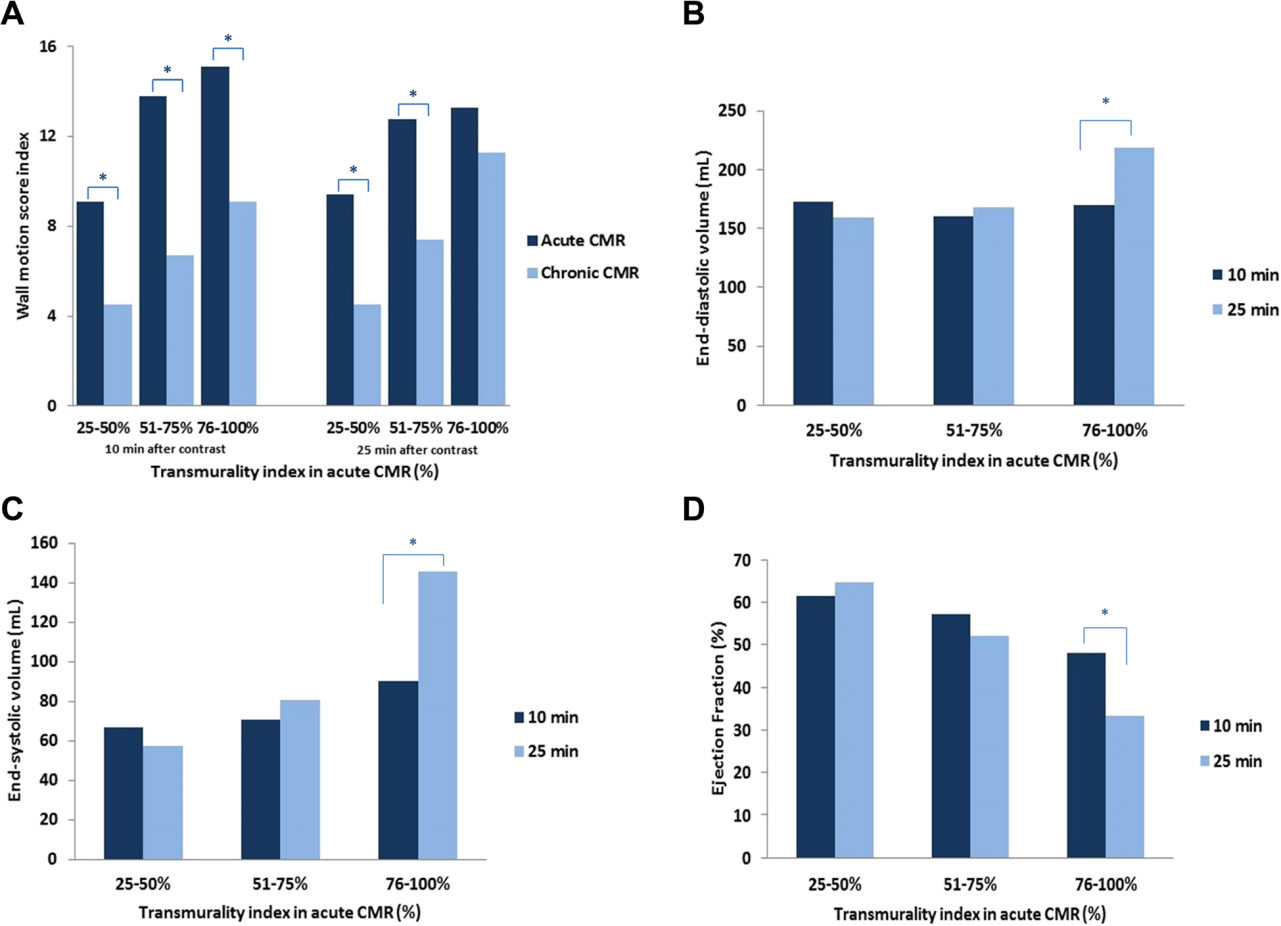
6**A**: Variation of the wall motion score index (WMSI) from acute CMR to chronic CMR according to the degree of transmurality (assessed 10 or 25 minutes after the contrast injection). Note that there is a significant improvement in the WMSI at follow-up except when the transmurality index is > 75% and assessed at 25 minutes. 6**B**/6**C**/6**D**: End-diastolic **(B)**, end-systolic **(C)** volumes and ejection fraction **(D)** at follow-up according to the degree of transmurality assessed in the acute CMR at 10 and 25 min post-contrast. Note that only patients with a transmurality index > 75% measured 25 minutes post-contrast present a significant increase in the end-diastolic and end-systolic volumes and a significant decrease in the ejection fraction at follow-up. rohandkumar1@ucla.edu

These dynamic changes were also observed in the myocardial segmental recovery of function. Thus, in patients with a transmural index > 75% 10 min post-contrast, 3.25 ± 2.76 segments improved their function and the wall motion score index improved 4.00 ± 4.78 in the chronic CMR. However, in patients with a transmural index > 75% 25 min post-contrast, only 2.00 ± 1.97 segments recovered its function and the wall motion score index improved only 2.00 ± 3.50 (p = 0.046 for segment recovery and p = 0.028 for the wall motion score index) (Fig. 6).

Finally, patients with a transmural index > 75% in the acute CMR 25 minutes post-contrast presented higher increase of EDV (p = 0.039), ESV (p < 0.001) and a higher decrease of EF (p < 0.001) at follow-up compared to those patients to whom the transmural index > 75% was determined 10 minutes post-contrast. (Table 4, Fig. 6).

**Table 4:** Variation of CMR variables at follow-up in patients with a transmurality index > 75% (in the acute CMR) at different minutes post-contrast

Time	N	Δ WMSI	Δ Num segments	Δ EDV (%)	Δ ESV (%)	Δ EF (%)
**1 min**	18 (30%)^1^	2.90 ± 3.60	2.30 ± 2.21	12.73 ± 21.99	13.21 ± 43.06	−4.48 ± 31.23
**3 min**	24 (40%)^1^	4.93 ± 4.68	4.90 ± 2.56	9.87 ± 22.78	3.44 ± 40.08	3.48 ± 28.79
**5 min**	17 (28.33%)^1^	4.20 ± 3.01	4.80 ± 1.85	15.67 ± 22.55	15.84 ± 45.52	−0.47 ± 33.72
**7 min**	17 (28.33%)^1^	4.12 ± 4.36	4.55 ± 2.31	14.01 ± 28.71	14.79 ± 44.85	−2.59 ± 33.49
**10 min**	15 (25%)^1^	4.00 ± 4.78	4.25 ± 2.76	14.64 ± 25.56	11.08 ± 40.57	−2.01 ± 33.66
**15 min**	13 (21.67%)^1^	3.60 ± 5.03	3.20 ± 2.59	17.47 ± 24.60	17.05 ± 40.38	−4.38 ± 36.16
**20 min**	10 (16.67%)^1^	2.60 ± 3.78	2.60 ± 1.67	20.47 ± 30.93	24.81 ± 45.67	−8.75 ± 39.56
**25 min**	7 (11.67%)^1^	2.00 ± 3.50	2.00 ± 1.97	22.72 ± 27.52	26.15 ± 40.31	−10.27 ± 40.40

N: expresses the number of patients with a transmurality index > 75% in the acute CMR. Δ: expresses the change of the variables from the acute to the chronic CMR.

Data expressed as mean and standard deviation.^1^Data expressed as mean (percentages).

Min: minutes. WMSI: Wall motion score index. Num: number. EDV: end-diastolic volume. ESV: end-systolic volume. EF: ejection fraction.

## Discussion

We report the first detailed time course study, examining LGE in the setting of an acute and chronic MI in the same cohort of patients treated with primary PCI for STEMI. More importantly, we found that the timing and delay of viability imaging acquisition, following contrast administration, is vitally important in predicting global and segmental left ventricular recovery. This dynamic distribution of LGE is consistent with previous experimental and clinical studies [5,10].

The main findings in our study are that 1) the distribution of Gadolinium within the infarcted area constitutes a dynamic process, with a maximum volume 3 minutes after contrast administration and subsequent decreases over time; 2) these changes are present in the acute setting but not in chronic MI; 3) changes in LGE size is due to a decrease in the transmural extension (within the area at risk) rather than the lateral borders; 4) dynamics of Gadolinium also occur if MVO is present, in this case, a slower rate of wash-out is observed.

### Kinetics of Gadolinium-DTPA in the infarct and the peri-infarct zone

Different mechanisms have been proposed to account for the Gd-DTPA enhancement patterns seen in reperfused infarcted tissue and the peri-infarct zone. First, there is an increase in the extracellular volume due to interstitial edema and/or loss of cell membrane integrity, which would increase the volume of distribution of Gd-DTPA. These findings were confirmed by Arheden et al., who found that the distribution volume was higher in the infarcted myocardium and intermediate in the peri-infarct zone compared to remote myocardium, and increased significantly with prolongation of the ischemic period [22]. Also, Klein et al. showed that the increase in the volume of distribution was the mechanism for an increase in necrosis signal intensity as the partition coefficient (λ) was elevated compared to remote myocardium [23].

Another hypothesis is that injured myocardium may also have abnormal Gadolinium wash-in and wash-out kinetics due to various factors (changes in coronary flow rates, capillary permeability, or functional capillary density). Slow wash-in, in reperfused infarcted regions, would lead to early low tissue contrast concentrations, whereas slow wash-out would eventually lead to higher contrast concentrations compared with normal tissue [18,7]. In this setting, Kim et al. found that in normal regions, contrast wash-in and wash-out were rapidly reaching a steady state within 2–3 minutes. Rim regions presented a significant delay in contrast kinetics compared to the normal, but not to the necrotic, myocardium. Contrast kinetics in core regions were so slow that signal intensity did not reach a steady state after 30 minutes [7].

The fact that Gadolinium accumulates in the peri-infarct zone of reversibly injured myocardium tissue could explain our findings. This hypothesis is supported by the fact that these dynamic changes are only seen in the acute setting, where edema and necrotic myocytes are present, and not in chronic infarcts with a dense collagenous matrix. Moreover, as we have demonstrated, the reduction of the infarct size is due to a decrease in the transmural extension rather than at the lateral borders after a coronary occlusion. Reimer and Jennings [24] and others [25] demonstrated that myocytes necrosis occupy the entire subendocardial surface of the area at risk and the irreversible injury progresses as a “wave-front” towards the subepicardium.

### Dynamic changes of infarct size and left ventricular function

Classical studies have shown that the transmural extent of infarction as defined by CMR predicts improvement in contractile function in acute and chronic MI [1,2]. However, discordant results have been reported. Using tagging sequences at baseline and during low dose dobutamine infusion, Kramer et al. [8] concluded that transmural LGE overestimated irreversible injury in acute MI. Also, Beek et al. [26] reported that 25% of segments with transmural LGE after an acute MI might potentially improve their function after 13 weeks. Similar findings have been reported by Dall’Armellina et al. [27], concluding that the acutely detected LGE does not necessarily equate with irreversible injury and may severely underestimate salvaged myocardium. In these studies, LGE was acquired 5–10 minutes after the contrast administration.

Although our and other studies [28,10,5] support the hypothesis that LGE can occur in reversibly-injured myocardial tissue in acute MI, studies in animals with acute MI have shown that LGE only occurs in irreversibly-injured tissue [3]. Close inspection of the methodology of these studies reveals some differences that should be considered: 1) Different sequences have been used to assess LGE images. 2) The time after contrast injection and image acquisition can vary from 5 to 30 minutes. 3) There can be differences between different species. Thus, Oshinski et al. found that the true infarct size, as assessed by TTC-staining, was overestimated by 20% to 40% immediately after contrast injection and that the time for the enhanced region to correspond to the true infarct size was at 21 ± 4 minutes [5].

Nevertheless, when the LGE is performed 20 minutes after the contrast injection the transmural extent of LGE correlated inversely with wall thickening and the ejection fraction both in the acute phase and at follow-up [29]. These results are concordant with our findings where using the transmurality index at 25 minutes correlated with a non-significant improvement of the WMSI and an adverse left ventricular remodeling at follow-up compared to patients with a transmural infarction estimated 10 minutes after contrast injection.

### Microvascular obstruction

MVO in LGE imaging has been reported to occur in 28% to 58% of patients after STEMI [17]. Klein et al. also found that the pattern of wash-in and wash-out was severely reduced in areas with marked MVO in images 18 minutes post contrast [23]. Similar findings were found in our study, thus, in patients with *late MVO*, dynamic changes of LGE were delayed compared to patients with smaller areas of MVO. Moreover, we demonstrate a rapid decline in the presence and volume of MVO in the minutes following contrast administration. This fact is of paramount importance as these rapid changes can potentially bias studies evaluating interventional trials aimed in reducing MVO. Nevertheless, due to the reduced number of patients in each group we could not find statistically significant differences and this finding should be further investigated.

### Delayed enhancement CMR sequences and technical aspects

The segmented technique has been used as the gold standard for the detection of myocardial infarction and accurate assessment of viability [13]. However, Huber et al. [12] described a new fast multislice technique, the single-shot sequence with an excellent correlation with the segmented to assess the size of the infarcted myocardium. This sequence allows imaging of nine slices during one breath-hold and, therefore, has the advantage of shortening scanning times, making it suitable for use with uncooperative patients.

A mixed T1 and T2 contrast is known for single-shot sequences [12]. As a result, the area of infarction could be overestimated in patients with an acute MI because of the edema and may justify the dynamic changes of LGE observed in *acute CMR*. Nevertheless, this hypothesis cannot be supported for many reasons: 1) the parameters used in the single-shot sequence (the inversion pulse, a short TR and TE, and a flip angle of 50°) support the T1 contrast compared with the T2 contrast. 2) The area of infarction is not overestimated on the ss-IR sequence when compared with the seg-IR at the same slice position.

The methodology used to measure LGE sequences is one of the most important points to consider in order to accurately quantify the size of MI. Kim et al. validated that LGE closely tracks the area of irreversible myocardial injury using an intensity thresholding of 2 SD above the mean of the normal myocardial intensity [3]. Although newer automated methods have been developed [30,31], there is no consensus for a precise method to automatically assess the real infarct size. In our study, we adopted the methodology of 2SD for both acute and chronic infarct mass, thus, the dynamic change of LGE volumes over time has been determined. Also, in our analysis, increasing the SD thresholds resulted in smaller infarct volumes in the same proportion for acute and chronic scars. However, the use of different SD is arbitrary and for that reason the use of T1 mapping sequences with a more quantitative assessment of the T1-values of the myocardium at different time points could potentially confirm more objectively our results.

### Limitations

Currently, the contrast kinetics following 25 minutes are unknown. However, due to the stability of infarct mass 20 to 25 minutes after the administration of the contrast and the specificity of the transmurality index at 25 minutes to predict the functional recovery at follow-up, we do not expect that longer periods of time would imply significant differences in our results.

Although our study suggests that the volume of LGE 3 minutes after contrast injection should correlate the AAR (early contrast enhancement) and the infarct size 25 minutes after (late contrast enhancement), we did not perform T2-weighted images to compare our findings. However, using the same patient population we have previously demonstrated a good correlation within the Infarct-ESA and the BARI score to assess the AAR [21].

## Conclusion

There is a change in LGE size over time in acute human reperfused STEMI not seen in the chronic phase, with a maximum size 3 minutes after the contrast administration and a decrease thereafter. This reduction occurs within the area at risk (transmural extension) rather than in the lateral boundaries.

It is important to wait at least 25 minutes to ensure a more accurate determination of infarct size in acute but not in chronic infarcts, in order to better predict left ventricular recovery at follow-up.

We consider that these findings have important implications and should be considered in clinical trials that consider infarct size and infarct transmurality as end-points or to predict global or regional recovery.

## Abbreviations

CMR: Cardiovascular magnetic resonance
LGE: Late Gadolinium enhancement
TTC: Triphenyltetrazolium chloride staining
MI: Myocardial infarction
STEMI: ST-segment elevation myocardial infarction
PCI: Percutaneous coronary intervention
LV: Left ventricle
EDV: End-diastolic volume
ESV: End-systolic volume
EF: Ejection fraction
WMSI: Wall motion score index
MVO: Microvascular obstruction
SD: Standard deviation
Gd-DTPA: Gadolinium-DTPA
SS-IR: Single-shot inversion recovery steady-state free precession sequence
Seg-IR: Segmented inversion-recovery gradient-echo sequence
Infarct-ESA: Infarct endocardial surface area
